# Supplementary material for: The causal relationship between the human gut microbiota and pyogenic arthritis: a Mendelian randomization study
Source: Front Cell Infect Microbiol. 2024 Nov 26;14:1452480. doi: 10.3389/fcimb.2024.1452480 (PMC11629706; doi:10.3389/fcimb.2024.1452480)
Supplement: Supplementary file 1 [file DataSheet1.zip › mendelian test/ebi-a-GCST90027588.csv_leave_one_out.pdf]

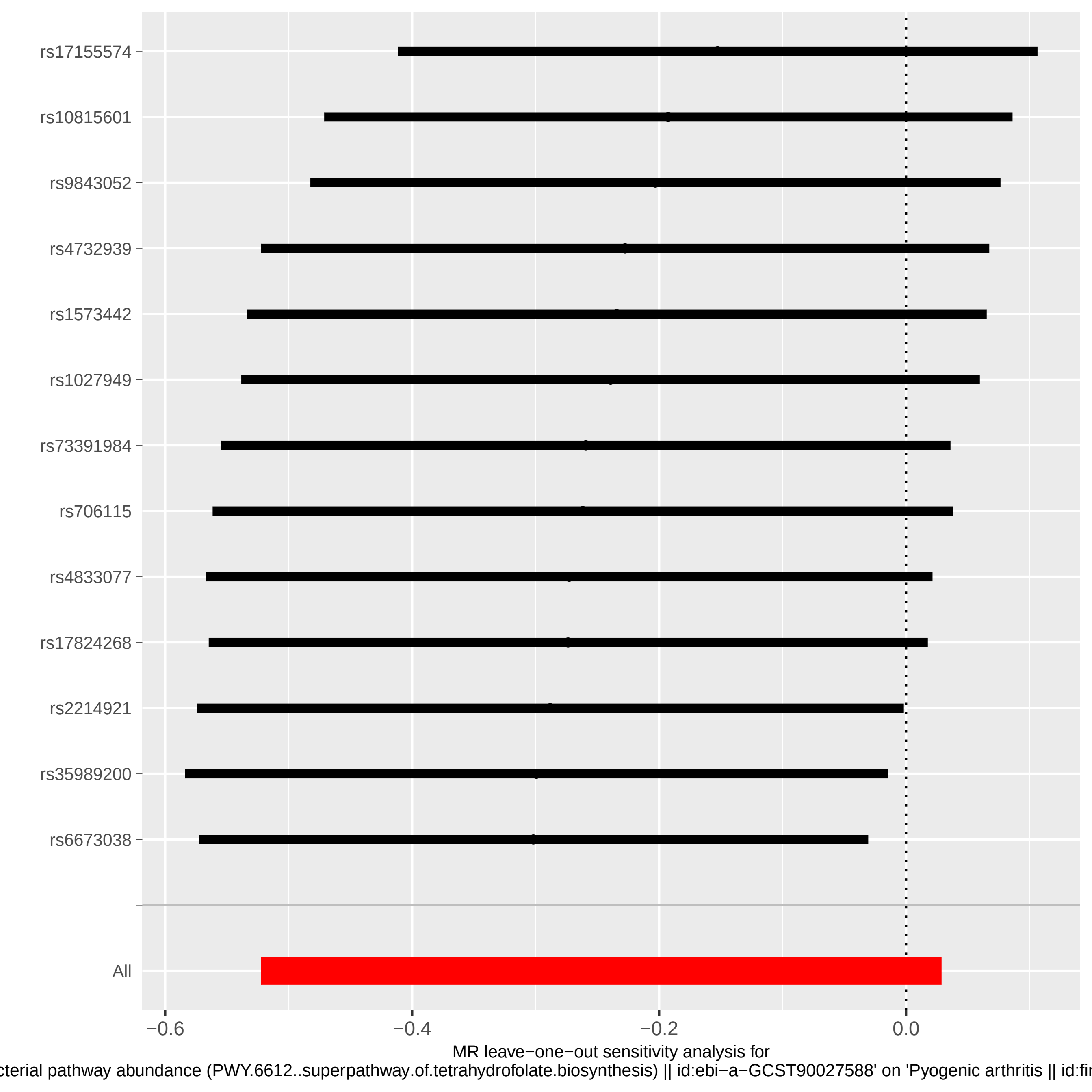

rs17155574
rs10815601
rs9843052
rs4732939
rs1573442
rs1027949
rs73391984
rs706115
rs4833077
rs17824268
rs2214921
rs35989200
rs6673038
All
−0.6
−0.4
−0.2
0.0
MR leave−one−out sensitivity analysis for
cterial pathway abundance (PWY.6612..superpathway.of.tetrahydrofolate.biosynthesis) || id:ebi−a−GCST90027588' on 'Pyogenic arthritis || id:fir
